# Effects of multi-walled carbon nanotubes (MWCNT) under *Neisseria meningitidis *transformation process

**DOI:** 10.1186/1477-3155-9-53

**Published:** 2011-11-16

**Authors:** Ives B Mattos, Danilo A Alves, Luciana M Hollanda, Helder J Ceragiogli, Vitor Baranauskas, Marcelo Lancellotti

**Affiliations:** 1 LABIOTEC - Biotechnology Laboratory, Department of Biochemistry, Institute of Biology CP6109, University of Campinas - UNICAMP 13083-970, Campinas, SP, Brazil; 2 NanoEng - NanoEngineering and Diamond Laboratory, School of Electrical and Computer Engineering, Department of Semiconductors, Instruments and Photonics, University of Campinas, UNICAMP, Av. Albert Einstein N.400, CEP 13 083-852 Campinas, São Paulo, Brasil

## Abstract

**Background:**

This study aimed at verifying the action of multi-walled carbon nanotubes (MWCNT) under the naturally transformable *Neisseria meningitidis *against two different DNA obtained from isogenic mutants of this microorganism, an important pathogen implicated in the genetic horizontal transfer of DNA, causing the escape of the principal vaccination measured worldwide by the capsular switching process.

**Materials and methods:**

The bacterium receptor strain C2135 was cultivated and had its mutant DNA donor M2 and M6, which received a receptor strain and MWCNT at three different concentrations. The inhibition effect of DNAse on the DNA in contact with nanoparticles was evaluated.

**Results:**

The results indicated an in increase in the transformation capacity of *N. meninigtidis *in different concentrations of MWCNT when compared with negative control without nanotubes. A final analysis of the interaction between DNA and MWCNT was carried out using Raman Spectroscopy.

**Conclusion:**

These increases in the transformation capacity mediated by MWCNT, in meningococci, indicate the interaction of these particles with the virulence acquisition of these bacteria, as well as with the increase in the vaccination escape process.

## Introduction

*Neisseria meningitidis *is a commensal bacterium of the human upper respiratory tract that may occasionally provoke invasive infections such as septicemia and meningitis. It is also naturally competent and therefore can exchange genetic information with each other by this process. This natural competence has been directly correlated to pilliation of these organisms, as well as a specific uptake sequence, within the genome of these bacterium [1].

The use of mutations for the study of the capsular polysaccharide of *N. meningitidi*s is the aim of several studies of the meningococci pathogenesis [2-4]. The capsular polysaccharide is the major virulence factor and a protective antigen. Meningococcal strains are classified into 12 different serogroups according to their capsular immune specificity, along with serogroups A, B, C, Y and W135 are the most frequently found in invasive infections. The capsule of serogroups B, C, Y and W135 strains is composed of either homopolymers (B and C) or heteropolymers (Y and W135) of sialic acid-containing polysaccharides that are specifically linked, depending on the serogroup [5,6]. This polymerization is mediated by the polysialyltransferase, encoded by the *siaD *gene in strains of serogroups B and C (also called *synD *and *synE*, respectively) and by *synG *in serogroup W135. Capsule switching after replacement of *synE*, in a serogroup C strain, by *synG *may result from the conversion of capsule genes by transformation and allelic recombination [7-10]. Such capsule switching from serogroup C to B *N. meningitidis *was observed in several countries, either spontaneously or after vaccination campaigns [7-13]. It might explain the emergence and the clonal expansion of strains of serogroup W135 of *N. meningitidis *in the year 2000 among Hajj pilgrims who had been vaccinated against meningococci of serogroups A and C [14]. These W135 strains belong to the same clonal complex ET-37/ST-11 as prominent serogroup C strains involved in outbreaks worldwide [8,9,15]. Hence, the emergence of these W135 strains in epidemic conditions raised the question about a possible capsule switching as an escape mechanism to vaccine-induced immunity. Also, these events are expected to occur continuously and can be selected by immune response against a particular capsular polysaccharide [9].

However, the interference of immune response with transformation efficacy has not been yet evaluated. Specific capsular antibodies are expected to bind to the bacterial surface and hence they interfere in DNA recognition and uptake. Also, environmental interference under the transformation process of this bacterium is unknown.

This work aimed at the use of multi-walled carbon nanotubes (MWCNT) for the study of the nanostructures action on the transformation process of meningococci, specifically their functions under the capsular switching process. The methods used in this work aimed at the action of MWCNT in the transformation of serogroup C *N. meningitidis *against two different DNA obtained from isogenic mutants of this microorganism.

## Methods

### Synthesis of multi-walled carbon nanotubes

**Figure 1:**
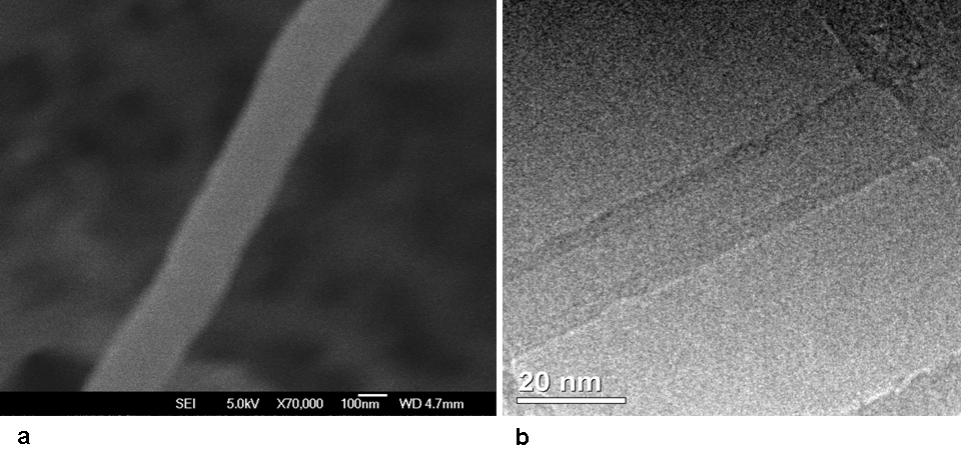
**shows (a) the morphologic by SEM and (b) structure by HRTEM of typical as deposited MWCNTs**. Scale bars are indicated, the outer diameter is ca. 200 nm and length > 1500 nm for the MWCNT show in (a). Multi-walled structures are presented in (b) corresponding to a MWCNT with outer diameter of ca. 20 nm.

The carbon nanotubes were produced by the process of hot filament chemical vapor deposition (HFCVD), at the Nanoengineering and Diamond Laboratory (NanoEng) of the Department of Semiconductors, Instruments and Photonics of the UNICAMP School of Electric Engineering and Computer Science. The carbon nanotubes were made in a copper substrate covered by a conductive polymer film called polyaniline. The polyaniline film covering the copper was dried on a hot plate at 100°C. After that, 0.2 ml of a 2 g/l acetone-diluted nickel nitrate (Ni(NO_3_)_2_) (where the nickel is the catalyzer for the growth of carbon nanotubes) was dropped on the dry polyaniline film. After drying, in room temperature, the polyaniline film was introduced into the HFCVD reactor in nitrogen atmosphere at 450°C and 27 mbar pressure for 30 minutes of growth time. An acetone solution of camphor bubbled in hydrogen gas was used as source of carbon. Morphological analyses were made by FESEM (Field Emission Scanning Electron Microscopy) using a JEOL JSM-6330F operated at 5 KV, 8 μA, and HRTEM (High Resolution Transmission Electron Microscopy) using a JEOL JSM 3010 operated at 300 KV and 73 μA. Figure 1 shows typical images of FESEM and HRTEM. We also used other nanostructures to confirm our results as the NC nanotubes (commercially obtained from Helix Material Solutions, USA), the NT2 were described by Grecco *et al*. [16].

### Bacterial Strains and Media

The characteristics of the strains used in this study are described in Table 1. They were grown at 37°C under 5% of CO_2 _on GCB agar medium (Difco) containing the supplements described by Lancellotti *et al*. [9]. When needed, culture media were supplemented with erythromycin at 2 μg/ml and spectomycin at 40 μg/ml. *Escherichia coli *strains used for plasmid preparations were DH5α [17].

**Table 1:** Bacterial Strains used in this work

Strain	Characteristics	Origin (Reference)
**DH5∝**	*Escherichia coli *F-, endA1, hsdR17 c, supE44, thi-1, gir A96, relA1	[17]

**pLAN45**	Plasmid containing *ΔNMB0065::ΩaaDA *derivated from pGEMTEasy	[18]

**pLAN13**	Plasmid containing the fusion of *synG::ermAM*	[18]

**C2135**	*Neisseria meningitidis *serogroup C, BIOMERIEUX	INCQS - FIOCRUZ

**W135ATCC**	*Neisseria meningitidis *serogroup W135, ATCC35559	INCQS - FIOCRUZ

**M2**	*N.meningitidis *isogenic mutant *ΔNMB0065::ΩaaDA*	[18]

**M6**	*N.meningitidis *W135ATCC transformed with pLAN13 to generate a fusioned strain *synG:ermAM*	[18]

### DNA Techniques

Recombinant DNA protocols and transformation were performed as described previously [18]. The oligonucleotides used are listed in Table 2. All the mutants obtained by homologous recombination were checked by Polymerization Chain Reaction - PCR analysis using an oligonucleotide harboring the target gene and another harboring the cassette.

**Table 2:** Oligonucleotides used in this work

Oligonucleotide	Sequence 5'-3'	Description
**03.12-3**	TGCGGATCCGCAGTAATTTTATCGGTTGG	NMB0065 forward
**03.12-4**	CCCCACTACCTAAAAAATGCTGATTTG	NMB0065 reverse
**aadA1**	TGCCGTCACGCAACTGGTCCA	Ω*aaDA *forward
**aadA2**	CAACTGATCTGCGCGCGAGGC	Ω*aaDA *reverse
**98.30**	GGTGAATCTTCCGAGCAGGAAA	*synG *forward
**98.31**	AAAGCTGCGCGGAAGAATAGTG	*synG *reverse
03.12-5*	TCGGGATCCTTATTTTTCTTGGCCAAAAA	*synG *reverse
**04.02-1**	CAATGAATCTCGCGTTGCTGTAGGTG	*synG *forward
**04.02-2**	GAAAAATAATTTGGGGCTTAGG	*synG *forward
**galECK29A**	CTTCCATCATTTGTGCAAGGCTGC	*galE *reverse
**ERAM1**	GCAAACTTAAGAGTGTGTTGATAG	*ermAM *forward
**ERAM3**	AAGCTTGCCGTCTGAATGGGACCTCTTTA GCTTCTTGG	*ermAM *reverse

*The underlined sequences in italic are the insertion of the *BamH*I site into original sequence

### Construction of NMB0065 mutant by polar mutation

**Figure 2:**
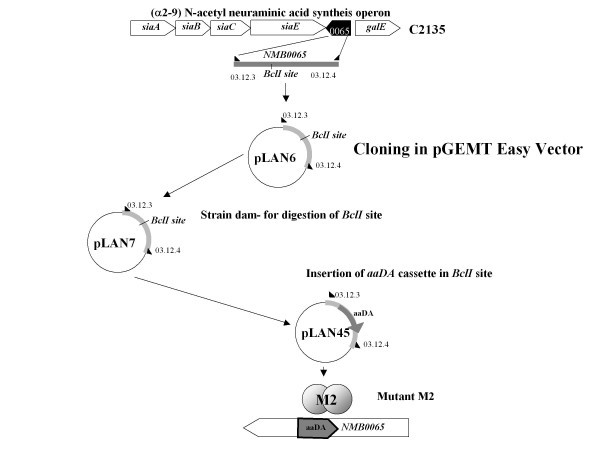
**shows schematic representation of the capsule genes of C serogroup in disrupted construction of NMB0065 gene with *aaDA *cassette**. The NMB0065 gene was amplified using the 03-12-3 and 03-12-4 oligonucleotides (table 3) from C2135 strain. This fragment was cloned into the pGEM-T Easy Vector System II (Promega Corporation, USA), to generate the plasmid pLAN6. *E. coli *strain Z501 was transformed with plasmid pLAN6 resulting in the plasmid pLAN7. The Ω*aaDA *cassette was inserted into the *BclI *site of pLAN7 to generate plasmid pLAN45, which was transformed into the C2135 strain to generate the isogenic mutant strain M2 [18,19].

This mutant construction follows the specifications described by Hollanda *et al*. [18]. Briefly, the NMB0065 sequence from *N. meningitidis *C2135 was amplified using 03.12-3 and 03.12-4 oligonucleotides (table 2). This fragment was cloned into the pGEM-T Easy Vector System II (Promega Corporation, Madison, WI, USA), to generate the plasmid pLAN6. *E. coli *strain Z501 was transformed with plasmid pLAN6 resulting in the plasmid pLAN7. The Ω*aaDA *cassette was inserted into the *BclI *site of pLAN7 to generate plasmid pLAN45, which was transformed into the C2135 strain, generating the mutant strain M2 (Figure 2).

### Construction of serogroup W135 mutants in transcriptional fusion *synG*::*ermAM*

**Figure 3:**
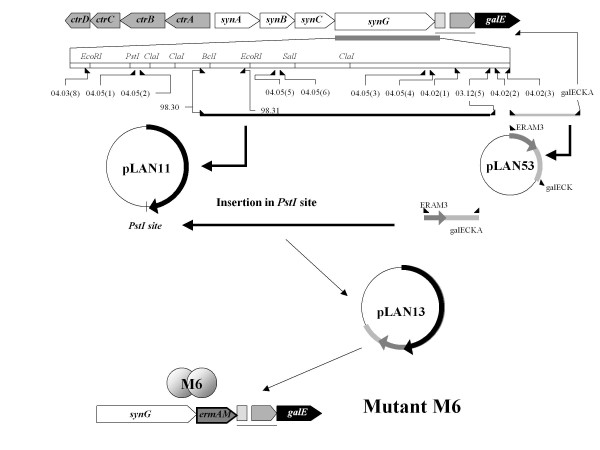
**Schematic representation of the capsule genes of W135 serogroup in transcriptional fusion of *synG *with *ermAM *cassette**. The *synG *gene responsible for the synthesis of the W135 capsule was amplified using the 98-30 and 03-12-5 oligonucleotides (table 2) from W135_ATCC _strain. The amplified fragment was cloned into the pGEM-T Easy Vector System I (Promega, Madison, WI, USA), to generate the plasmid pLAN11. In the same conditions, another fragment was amplified using the 04.02-2/galECK29A from *synG *downstream sequence to generate pLAN52. The *ermAM *cassette was inserted into *Nco*I site of pLAN52 to generate pLAN53. The fragment amplified from pLAN53 with the ERAM1 and galECK29A [19] was inserted into *Pst*I site of pLAN11 to generate pLAN13-2. This plasmid was linearised by the enzyme SphI and transformed into W135_ATCC _strain to generate the *synG::ermAM *strain M6, erythromycin resistant [18].

As the mutant M2, this mutant construction follows the specifications described in Hollanda *et al*. [18]. Briefly, the *synG *gene responsible for the synthesis of the W135 capsule was amplified using the 98-30 and 03-12-5 oligonucleotides (table 2) from the serogroup W135 strain W135ATCC. The amplified fragment was cloned into the pGEM-T Easy Vector System I (Promega, Madison, WI, USA), to generate the plasmid pLAN11 (Figure 3). Another fragment was amplified using the 04-02-2/galECK29A from *synG *downstream sequence, cloned into pGEM-T Easy Vector, to generate pLAN52. The *ermAM *cassette was amplified by ERAM1/ERAM3 and inserted into *Nco*I site of pLAN52 to generate pLAN53. The fragment amplified from pLAN53 with the ERAM1 and galECK29A [19] was inserted into *Pst*I site of pLAN11 to generate pLAN13-2. This plasmid was linearised by the enzyme SphI and transformed into W135_ATCC _strain to generate the *synG::ermAM *fusioned strain M6, erythromycin resistant.

### Analysis of transformation frequency up to MWCNT contact

At 1.10^8 ^colony-forming units - CFU - of the receptor strain C2135, we added 1 μg genomic DNA from M2 and M6 mutants and 10, 20 and 50 μg of different MWCNT. A negative control was also performed without MWCNT. The suspension was incubated for three hours at 37°C in atmosphere of 5% of CO_2 _by three hours. The counts of total CFU were performed in GCB spectinomycin or erythromycin plates in triplicate analysis (for M2 and M6 isogenic mutants, respectively). The CFU obtained in plates containing specific antibiotic were analyzed by PCR for the presence of target gene transfer in the transforming units (Ω*aaDA *cassette for the M2 DNA and *synG *for M6 donor DNA). In order to verify the interaction between DNA, MWCNT and DNAse action, the same amounts of DNA(1 μg) from M2 and M6 mutants, MWCNT (20 μg) and bacterial cells were submitted to action 5 U of DNase (New England Biolabs, UK) and further transformation process. Also, the counts of cfu were performed in GCB spectinomycin or erythromycin plates in triplicate analysis (for M2 and M6 isogenic mutants respectively).

### Analysis of interaction between DNA and MWCNT by Raman spectroscopy

The prior analysis of DNA from M2 and M6 mutant strains with MWCNT was performed under a mix of 1 μg of M6 genomic DNA and 20 μg of MWCNT. The samples were characterized by Raman spectroscopy [20,21]. The spectra were recorded at room temperature using a Renishaw microprobe in Via system, employing an UV laser for excitation (λ = 325 nm) at about 10 mW. The samples M2 and M6 were dripped onto a quartz substrate for UV laser Raman spectroscopy.

## Results and discussion

**Figure 4:**
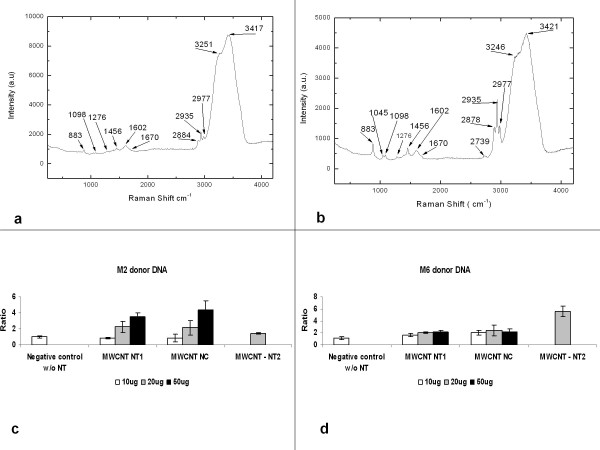
**shows signal of DNA in the region until 2000 cm^-1^**. DNA consist of three groups: phosphates, deoxyribose and four bases such as A (adenine), T (thymine), C (cytosine) and G (guanine). In our work, the bands could be assigned to 883/1098/1045 cm^-1 ^- O-P-O backbone; 1276 cm^-1 ^- C (cytosine); 1456 cm^-1^- A (adenine);1602 cm^-1 ^- guanine (G) and 1670 cm^- ^- T (thymine). The bands of DNA donor M2 (a) and M6 (b) are in accordance with some authors [21,22]. The bands 2739-3421 cm^-1 ^are not assigned to DNA, it is assigned to the quartz substrate. In (c) and (d) it shows the action of the MWCNT under *Neisseria meningitidis *strain C2135 using as donor DNA the M2 (c) and M6 (d).

The effects of the MWCNT were verified by an increase in the number of CFU obtained from many transformation processes. The CFU number resulting from the transformation process using DNA from M2 donor strain was higher than the one obtained using M6 as the donor strain. Also, the use of three different MWCNT and three different concentrations (10, 20 and 50 μg of each MWCNT) showed an increase in the number of CFU resulted from the transformation process using both DNA donor strains (Figure 4(c-d) and table 3).

**Table 3:** Values obtained from C21 35 transformation using the donor DNA from M2 and M6 mutants.

Donor DNA (1 μg)	Ratio (means obtained exposed to MWCNT/mean of negative control)	*P *values (one way Tukey's test)
**Negative Control (without MWCNT) M2**	1.02 ± 0.17	
**NT1 (10 μg)**	0.89 ± 0.09	*P *= 0, 1631 (non significant)
**NT1 (20 μg)**	2.24 ± 0.70	*P *< 0, 05 (P = 0, 0496 significant)
**NT1 (50 μg)**	3.52 ± 0.50	*P *< 0, 05 (P = 0, 0073 very significant)
**NC (10 μg)**	0.85 ± 0.50	*P *= 0, 3166 (non significant)
**NC (20 μg)**	2.18 ± 0.90	*P *= 0, 0798 (non significant)
**NC (50 μg)**	4.36 ± 1.18	*P *< 0, 05 (P < 0, 0020 significant)
**NT2 (20 μg)**	1.42 ± 0.13	*P *< 0, 05 (P = 0, 0240 significant)

**Negative Control (without mesoporous siliM6**	1.09 ± 0.25	
**NT1 (10 μg)**	1.71 ± 0.25	*P < 0, 05 *(P = 0, 0385 significant)
**NT1 (20 μg)**	2.03 ± 0.08	*P < 0, 05 *(P = 0, 0034 very significant)
**NT1 (50 μg)**	2.11 ± 0.30	*P < 0, 05 *(P = 0, 0106 significant)
**NC (10 μg)**	2.03 ± 0.35	*P < 0, 05 *(P = 0, 0193 significant)
**NC (20 μg)**	2.44 ± 0.88	*P < 0, 05 *(P = 0, 0490 significant)
**NC (50 μg)**	2.14 ± 0.49	*P < 0, 05 *(P = 0, 0403 significant)
**NT2 (20 μg)**	5.58 ± 0.86	*P < 0, 05 *(P = 0, 0065 very significant)

The intention of two different DNA donors was to certificate the independence of MWCNT action under the same bacterial strain - *N. meningitidis *C2135. Further analysis by PCR demonstrated the transfer of the tagged gene from M2 and M6 in transformed strains (data not shown). The Raman analysis showed the interaction of MWCNT with the DNA obtained from M6 mutant strains as viewed in Figure 4(a-b).

Data analyses were made by ratio values between the numbers of transformants cfu obtained with MWCNT by median values of transformants cfu obtained without nanotubes treatment (Figures 4c-d and table 3). The values were analyzed by one-way analysis of variance ANOVA (Tukey's test compared each treatment to control without nanoparticles in transformation, considering significant values of P > 0.05). Some values obtained with commercial MWCNT - NC and NT2 showed different results when compared with NT1 (table 3 and Figure 4).

The relations between the meningococci transformation and MWCNT action viewed in these results could mimic the presence of carbon nanoparticles in atmosphere and evoke the emergence of outbreaks of Brazilian purpuric fever (BPF) caused by another naturally competent bacteria, *Haemophilus influenzae *biogroup *aegyptius *[22,23]. The *Haemophilus influenzae *biotype *aegyptius *causes BPF, a dangerous inflammatory disease known as *purpura fulminans *with a great mortality rate [24]. Kroll *et al*. [24] described these *Haemophilus influenzae *strains, usually associated with conjunctivitis cases, as a product of horizontal transfer between *N. meningitidis *and *Haemophilus influenzae*. In the same geographic region of these outbreaks, the primitive agricultural practice, performed by burning sugar cane, generates an emission of carbon micro and nanoparticles in the atmosphere, potentially provoking respiratory disorders by particles inhalation [25]. Our group has been studying these bacteria and testing them with MWCNT on its transformation process.

This process is similar to the phenomena of capsular switching as described in sub Saharan African [26-28] and Saudi Arabian regions (Hajj pilgrimage) [26,29-35]. In desert zones, the *ramarthan *wind and the presence of silica nanostructures facilitates the capsular switching process in meningococci strains [26,29-36]. Thus, new experiments using animal models that could confirm this hypothesis have been performed by our group. Also, the increases in the transformation capacity in bacteria have been verified in *Escherichia coli *by nanotube structures, as described by Rojas-Chapana *et al*. [37].

**Figure 5:**
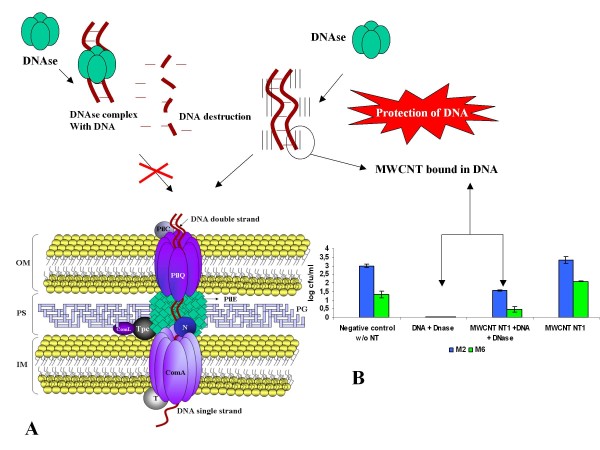
**Schematic representation of the inhibitor effect of the DNase by MWCNT**. (a) the transformation bacterial complex formed by many proteins as pilQ, pilE, ComA, and accessory proteins distributed around the outer membrane (OM), periplasmatic space (PS) and inner membrane (IM) in naturally transformable Gram negative bacteria, specially *Neisseria *species [38]. (b) Graph showing the inhibitory effect of MWCNT in DNase avoiding the DNA lyses.

The results of DNAse inhibition over free DNA (Figure 5) could explain the protection of the bacterial genes by MWCNT contact in this nanostructure. This evidence is showed in the graphic of Figure 5 with the increase of CFU in the test containing DNAse-treated DNA and exposed to 20 μg of MWCNT. These results need further experiments in order to better understand this interaction between bacterial compounds and the transformation system (represented in Figure 5). Furthermore, animal models, for these studies, may be very interesting for future assessments of atmospheric contamination by carbon nanoparticles produced by primitive agriculture and carbon miners.

This work indicated, for the first time in scientific literature, that the action of atmospheric nanoparticles obtained from anthropic activities, such as primitive agriculture, influences the bacterial transformation process.

## Conclusion

The increase in the transformation capacity mediated by MWCNT in meningococci indicates an interaction of these particles with the bacterial DNA leading to virulence acquisition and an increase in the escape to vaccination. The presence of these nanoparticles protects the DNA from DNAse action, increasing the recombination frequency. These results show that important measures for public health, in places where the MWCNT or carbon microparticles are produced, need to be carefully revised.

## Competing interests

The authors declare that they have no competing interests.

## Authors' contributions

**IBM **carried out the Molecular Biology design and plasmids; **DAA **carried out the molecular microbiologic tests; **LMH **carried out the molecular genetics studies; **HJC **carried out the MWCNT synthesis and FESEM, HRTEM and Raman tests; **VB **participated in the drafting of the manuscript and gave technical support in Nanoengineering; **ML **carried out the molecular genetics studies and also the draft of the manuscript. All the authors read and approved the final manuscript.
